# PON-All: Amino Acid Substitution Tolerance Predictor for All Organisms

**DOI:** 10.3389/fmolb.2022.867572

**Published:** 2022-06-16

**Authors:** Yang Yang, Aibin Shao, Mauno Vihinen

**Affiliations:** ^1^ School of Computer Science and Technology, Soochow University, Suzhou, China; ^2^ Collaborative Innovation Center of Novel Software Technology and Industrialization, Nanjing, China; ^3^ Department of Experimental Medical Science, Lund University, Lund, Sweden

**Keywords:** variation interpretation, mutation, animal variants, plant variants, amino acid substitution, prediction, pathogenicity, machine learning

## Abstract

Genetic variations are investigated in human and many other organisms for many purposes (e.g., to aid in clinical diagnosis). Interpretation of the identified variations can be challenging. Although some dedicated prediction methods have been developed and some tools for human variants can also be used for other organisms, the performance and species range have been limited. We developed a novel variant pathogenicity/tolerance predictor for amino acid substitutions in any organism. The method, PON-All, is a machine learning tool trained on human, animal, and plant variants. Two versions are provided, one with Gene Ontology (GO) annotations and another without these details. GO annotations are not available or are partial for many organisms of interest. The methods provide predictions for three classes: pathogenic, benign, and variants of unknown significance. On the blind test, when using GO annotations, accuracy was 0.913 and MCC 0.827. When GO features were not used, accuracy was 0.856 and MCC 0.712. The performance is the best for human and plant variants and somewhat lower for animal variants because the number of known disease-causing variants in animals is rather small. The method was compared to several other tools and was found to have superior performance. PON-All is freely available at http://structure.bmc.lu.se/PON-All and http://8.133.174.28:8999/.

## Introduction

Genome and exome sequencing are frequently used techniques in biology and clinical settings. Efficient resequencing has moved the bottleneck from obtaining sequence and variation information to variation interpretation. Many tools have been released for variant pathogenicity, also called variant tolerance and prediction (Adzhubei et al., 2010; Choi et al., 2012; Olatubosun et al., 2012; Capriotti et al., 2013; Kircher et al., 2014; Schwarz et al., 2014; Dong et al., 2015; Niroula et al., 2015; Vaser et al., 2016; Rogers et al., 2018). These methods are also used for clinical diagnosis in many countries and laboratories according to American College for Medical Genetics and Genomics (ACMG) and the Association for Molecular Pathology (AMP) (Richards et al., 2015) guidelines. These guidelines state that predictions could support the diagnosis if several methods agree. This recommendation is problematic and should be reconsidered as it reduces the number of cases that can be predicted because the method with the poorest performance dictates the outcome (Vihinen, 2020).

Variant interpretation methods have been divided into three main categories: those based on evolutionary information, those utilizing many types of features, including evolutionary details, and meta-predictors that use predictions from other predictors as the starting point (Niroula and Vihinen, 2016). Methods in the last two categories are typically based on machine learning (ML). Several different ML algorithms have been applied, there is not a single best one among them. The predictor performance depends on the quality of data, used features and their selection, implementation of the predictor, and other factors.

Most pathogenicity/tolerance predictors classify variants into two classes (pathogenic/benign), while some have three or more categories. Additional categories could be useful if the predictions are reliable because diseases are not simple binary states, as indicated by the pathogenicity model (Vihinen, 2017). These tools do not explain the cause and mechanism of diseases due to harmful variants. Many other types of predictors are available for various effects and mechanisms, including RNA splicing, protein stability, solubility, disorder, aggregation, and localization.

In variation interpretation, most of the work has been devoted to explaining human variants; however, there is increasing interest and need for interpretation of variants and their consequences also in other organisms. This knowledge is essential for understanding diseases in non-human organisms, obtaining insight into genetic disease mechanisms, genetic diagnosis in veterinary and botany, and scientific inquiry and comparison, among others. Although several predictors trained on human data are applicable to (or at least capable of) accepting variants from other organisms, they have not been systematically developed and tested for alterations from other organisms. Evolutionary methods could be easily adapted for this purpose. However, evolutionary data alone are of limited significance as they do not allow the development of the most reliable predictors. Variation interpretation is a very complex problem, and many features are needed to achieve high prediction performance.

Some predictors have been developed and trained on plant (Kono et al., 2018; Kovalev et al., 2018) and animal variant data (Plekhanova et al., 2019). In animal and plant experiments, it would be important to know whether the used strains contain harmful variants since they may act as confounding factors in various studies. In veterinary medicine, there is increased interest in variants (e.g., in pet animals) also outside the most common species of cats and dogs. Variation data and even genetic data are scarce for many of these species. Experimental validation of variation effects is laborious and often outside the available resources. Therefore, in many cases, the only means to assess the harmfulness of identified variants is to perform computational predictions. As there are not many special tools and even those available as generic methods have not been systematically tested, there is no way of knowing the reliability of the predictions. Some databases, especially the Online Mendelian Inheritance in Animals (OMIA), are valuable. However, there are currently data only for nine named species (and others). Further, the number of likely disease-causal variants is only 1,381. The best performing human variant effect predictors have been trained on tens of thousands of variants.

We have developed several methods for variation interpretation, mainly based on ML. These include PON-P (Olatubosun et al., 2012) and PON-P2 (Niroula et al., 2015) for human pathogenicity prediction of amino acid substitutions, PON-Tstab (Yang et al., 2018) for variants affecting protein stability, PON-Sol (Yang et al., 2016) and PON-Sol2 (Yang et al., 2021) for solubility affecting variants, PON-Diso (Ali et al., 2014) for protein disorder affecting alterations, and PON-mt-tRNA (Niroula et al., 2016) for variants in mitochondrial tRNA molecules. These tools are highly accurate and among the best in their application areas. Several aspects have to be considered in method development: data collection, feature selection, method training, and systematic performance benchmarking (Niroula and Vihinen, 2016).

We collected a data set of human, animal, and plant variants and trained an ML predictor using a gradient boosting algorithm and exhaustive feature selection. The method is called PON-All as it can predict the consequences of amino acid substitutions in proteins from any organism. Several predictors were developed and extensively tested by reporting a full set of performance measures. PON-All was systematically trained and tested and found to have very high performance in predictions for all three types of organisms. The method is fast and freely available as a web resource.

## Materials and Methods

### Data Sets

Amino acid substitutions in human, animal, and plant sequences were collected from databases and publications. The human variants were obtained from VariBench (Nair et al., 2013; Sarkar et al., 2020), including 13,885 harmful variants originally used to train PON-P2 (Niroula et al., 2015). Additional 6369 verified clinical cases were obtained from ClinVar (Landrum et al., 2014) and 2,058 variants in membrane proteins (Orioli and Vihinen, 2019) from VariBench. Only amino acid substitutions with harmful clinical effects were collected. Duplicate cases were removed.

Human neutral variations with minor allele frequency (MAF) 1%<MAF<25% were from ExAC and obtained from VariBench (http://structure.bmc.lu.se/VariBench/ExAC_AAS_20171214.xlsx). The data set had originally been used to test the sensitivity of several predictors (Niroula and Vihinen, 2019). Because these variants have high MAF in populations, they are considered benign. Benign variations used for training and testing were randomly selected. The numbers of variations used in different stages are indicated in Table 1. An additional set of 370 benign variants obtained from ClinVar was used to assess specificity.

**TABLE 1:** Division of cases to data sets for cross-validation and blind testing. The first number is for proteins and the second for variants.

	10-fold cross-validation			Blind test			Total		
	Pathogenic	Neutral	Total	Pathogenic	Neutral	Total	Pathogenic	Neutral	Total
Humans	2,173/17,504	12,141/23,600	13,383/41,104	170/1,980	669/1,967	740/3,926	2,343/19,484	12,810/25,567	14,123/45,030
Animals	117/162	116/144	232/306	109/155	125/169	233/324	226/317	241/313	465/630
Plants	913/2,601	629/1,562	1,150/4,163	228/736	152/374	288/1,110	1,141/3,337	781/1,936	1,438/5,273
Total	3,203/20,267	12,886/25,306	14,765/45,573	507/2,871	946/2,510	1,261/5,360	3,710/23,138	13,832/27,816	16,026/50,933

There were two sources for variations in animals. Cases with the notation “likely causal variants” were obtained from OMIA (Nicholas, 2003). Additional mammalian deleterious variants were obtained from Plekhanova et al. (2019). The main species included dogs, mice, and cattle. Plant data were taken from the data set used to develop a random forests pathogenicity predictor for plant protein variations (species included *Arabidopsis*, *Oryza sativa*, and *Pisum sativum*) (Kovalev et al., 2018). Altogether, there were 23,138 pathogenic variations and 27,816 neutral variations in 16,026 proteins in the three types of species.

As some features used in training were protein-specific, it was necessary to partition the cases so that all variants in the same protein were in the same data set (either training or test set) to ensure the universality of the classification and avoid bias. Further, we balanced the numbers of pathogenic and neutral cases.

The blind test data set contained cases used to test PON-P2 (Niroula et al., 2015). Half of the animal variants were randomly distributed to the blind test set. Because there were substantially more plant variants, we randomly selected 20% of the variants for the blind test set. In addition, the division of 10-fold cross-validation (CV) training sets and blind test sets ensured that the variations in any protein were always in either the training set or blind test set. Another principle of data division was that the numbers of harmful and neutral variations in each data set were balanced (1:1). The data sets used for training and testing are available in VariBench (Nair et al., 2013; Sarkar et al., 2020) at http://structure.bmc.lu.se/VariBench/trainingall.php and http://8.133.174.28:8999/.

### Features

To train the predictor, we started with 1,085 features: 617 amino acid features, 436 variation type features, 25 neighborhood features, 2 evolutionary conservation features, 1 protein feature, and 1 GO feature.

A total of 617 complete amino acid propensity scales were from AAindex (Kawashima and Kanehisa, 2000). This feature set has been previously used to train PON-P2 (Niroula et al., 2015), PON-PS (Niroula and Vihinen, 2017), PON-Tstab (Yang et al., 2018), and PON-Sol2 (Yang et al., 2021). For each variant, the difference between the score for the original amino and the variant amino acid was calculated.

There were two matrices to obtain variation-type features. A total of 400 features came from the 20*20 matrix, where the two dimensions represented original and variant residues. Another 36 features denote a 6*6 matrix representing the physical and chemical properties of amino acids. The six amino acid categories were hydrophobic (V, I, L, F, M, W, Y, C), negatively charged (D, E), positively charged (R, K, H), conformational (G, P), polar (N, Q, S), and others (A, T) and have been previously described (Shen and Vihinen, 2004).

In order to represent the sequence context of variation sites, 25 neighborhood features were included. A 20-dimensional vector of neighborhood residues counts the occurrences of each amino acid type within a neighborhood in a window of 23 positions, that is, 11 positions before and after the variation site (Lockwood et al., 2011). In addition, we included the frequencies of five groups of amino acids (nonpolar, polar, charged, positively charged, and negatively charged) in the neighborhood window of 23 positions.

For evolutionary conservation, DIAMOND (Buchfink et al., 2015) was used to compare each protein sequence to SwissProt (Shomer, 1997) to find related sequences and calculate the number of hits. DIAMOND was chosen as it is substantially faster than BLAST (Altschul et al., 1997) but with a similar degree of sensitivity. The identified sequences were aligned and then used to calculate SIFT scores for evolutionary conservation of each variant position using SIFT 4G (Vaser et al., 2016).

The protein feature was defined as the length of the protein sequence. Additional features included whether the variation was in the first amino acid in the peptide chain and position within the sequence.

Features derived from Gene Ontology (GO) terms have previously been used for variant classification (Kaminker et al., 2007; Calabrese et al., 2009; Niroula et al., 2015). For the full set of GO terms, we combined results from AmiGO (Carbon et al., 2009) and QuickGO (Munoz-Torres and Carbon, 2017) using the R Bioconductor tool GO.db (https://bioconductor.org/packages/GO.db/). We collected all the ancestors of all GO terms and filtered the GO entries so that each protein contained each GO term once. Two sets of GO terms were created for each category (pathogenic and neutral). The sum of the logarithm ratio of GO frequencies of the pathogenic set and that of the neutral set was calculated as follows:

$$LR\;=\sum \log\frac{f\left(P_{i}\right)\mathrm{+1}}{f\left(N_{i}\right)\mathrm{+1}},$$

where *LR* is the value for the GO annotations and *f*(*P*_*i*_) and *f*(*N*_*i*_) are the frequencies of the *i*th GO term in pathogenic and neutral data sets, respectively. To avoid uncertain ratios, we added 1 to all the frequencies. If a protein had not been annotated with GO terms, then *LR = 0*, and this feature was not considered in the prediction. We separately trained predictors with and without GO annotations.

We tested the usefulness of functional annotation features and found that almost all the variation records had functional annotations. Site-specific annotations were determined from UniProtKB/Swiss-Prot. The variations that occurred at such sites were identified. We collected all site terms and filtered them so that each protein contained each site term once. Two sets of site terms were created for the two categories (pathogenic and neutral). The sum of the logarithm ratio of site frequencies of the pathogenic set and that of the neutral set were defined as follows:

$$FS\;=\sum \log\frac{f\left(P_{i}\right)\mathrm{+1}}{f\left(N_{i}\right)\mathrm{+1}},$$

where *FS* is the value for the site annotations and *f*(*P*_*i*_) and *f*(*N*_*i*_) are the frequencies of the *i*th site term in pathogenic and neutral data sets, respectively. To avoid uncertain ratios, we added 1 to all the frequencies. If a protein had not been annotated with site terms, then *FS = 0,* and this feature was not considered in the prediction.

### Algorithms

We trained predictors with three machine learning algorithms: random forests (RF) (Breiman, 2001; Pavey et al., 2017), XGBoost (Chen et al., 2016; Yu et al., 2020), and Light GBM (LGBM) (Wang et al., 2017; Zhang et al., 2019). The default parameters were used in each case. All the algorithms were implemented in Python in the standard learn package (Pedregosa et al., 2011). Random forests is an ensemble algorithm. It applies several decision trees on a subset of the data set and uses the average accuracy of each decision tree to improve the performance and reduce overfitting. The gradient boosting model evaluates the output features based on the combination output result of weak prediction learner models. It minimizes a loss function to optimize the model. Sequential models are constructed using the decision trees until maximum accuracy is achieved.

XGBoost and LightGBM are implementations of gradient boosting. Initial results for LightGBM and XGBoost were similar and better than for random forests. Because of the similar performance, we chose LightGBM which is faster due to Gradient-based One-Side Sampling (GOSS) and Exclusive Feature Bundling (EFB) (Ke et al., 2017).

### Reliability Assessment

The probability method was used to identify variations with high confidence. The probability distribution function of self-sampling probability cannot be determined. Therefore, we used Chebyshev’s inequality, which is applicable to arbitrary distributions. For random variables *X* with mean µ and standard deviation σ, Chebyshev’s inequality guarantees that at least 1 − (1/*k* ^2^) values are distributed within *k* standard deviations of the mean values:

$$P\left(\mu -k\sigma <X<\mu +k\sigma \right)\geq 1-\frac{1}{k^{2}}.$$

When 1 − (1/*k* ^*2*^) is 0.95, and if the range of µ±*k*σ does not include 0.5, the prediction is marked as credible and classified as pathogenic or neutral; else, the variation is considered as unclassified (UV, unclassified variant, also called VUS, variant of uncertain significance).

### Performance Assessment

We used eight measures to evaluate the classification performance (Vihinen, 2012; Vihinen, 2013). The measures included positive predictive value (PPV), negative predictive value (NPV), sensitivity, specificity, accuracy, Matthews correlation coefficient (MCC) and overall performance measure (OPM) (Niroula et al., 2015). The mathematical definitions of these measures are as follows:

$$PPV=\;\frac{TP}{TP+FP}\;,$$

$$NPV=\frac{TN}{TN+FN}\;,$$

$$Sensitivity=\;\frac{TP}{TP+FN}\;,$$

$$Specificity=\frac{TN}{TN+FP}\;,$$

$$Accuracy=\frac{\;TP\;+\;TN}{TP\;+\;TN\;+\;FP\;+\;FN}\;,$$

$$MCC\;=\;\frac{\;\left(TP\times TN\right)-\left(FP\times FN\right)}{\sqrt{\left(TP+FN\right)\times \left(TP+FP\right)\times \left(TN+FN\right)\times \left(TN+FP\right)}},$$

$$nMCC=\frac{1+MCC}{2},$$

$$OPM=\;\frac{\left(PPV+NPV\right)\left(Sensitivity+Specificity\right)\left(Accuracy+nMCC\right)}{8}.$$

The area under the curve (AUC) was calculated from the Receiver Operating Characteristics (ROC) curve, where cases are plotted based on sensitivity *versus* 1 − specificity.

TP and TN are the numbers of correctly predicted pathogenic and neutral cases, and FN and FP are the numbers of wrong predictions for pathogenic and neutral cases, respectively.

Coverage measures the ratio of predicted cases among all the instances. *X* indicates the number of cases classified as harmful or neutral, and *Y* is the total number of test variants:

$$Coverage\;=\;\frac{X}{Y}.$$

The reason to measure coverage in this way is that PON-All classifies cases into three categories while the data for training and testing are binary (benign/pathogenic).

### Feature Selection

We used the recursive feature elimination (RFE) method (Guyon et al., 2002) to carry out multiple rounds of training. First, the prediction model was trained with all the features, and each feature was assigned a weight. Then, the features with the minimum absolute weight were removed. Recursion was repeated until the preset number of features was achieved. To identify the optimal set of features, we trained methods in addition to the full set of features also with 100, 50, 20, and 10 features.

## Results

There is an increasing interest in variation interpretation in several organisms. Many of the current variant tolerance/pathogenicity predictors are either just for humans or have not been systematically benchmarked and/or trained with data from other organisms than human. Therefore, we developed a PON-All tool to predict the consequences of amino acid substitutions in any organism. The method was trained on human, animal, and plant variants with known outcomes, validated on a blind test set, and compared to several other tools. The training and assessment of the performance are described according to published guidelines (Vihinen, 2012; Vihinen, 2013), the method is freely available, and the used data are distributed.

Variations were collected from several sources; see Table 1. There is a severe imbalance in the number of variants from different sources. The number of animal variants is clearly smaller than the others. There were 630 variants in 465 proteins in the animal data, while the corresponding numbers were 45,030 variants in 14,123 proteins in human and 5,273 variants in 1438 plant proteins (Table 1). The ratio of human, plant, and animal variants was 90:10:1.

The largest numbers of disease-causing animal variations are known in rodents (mice and rats). However, we excluded these variants because they are typical models for human diseases and the corresponding variants are largely included in the human data set. Further, using these cases in the blind test could biased the analysis. The included animal variants originate from animal conditions.

In the case of animal variants, we selected a substantially larger ratio to the test set to facilitate reliable performance assessment. Totally, we had 50,933 variants in 16,026 proteins, thus covering a wide spectrum of different sequences. A total of 23,138 of the variants were pathogenic and 27,816 were benign.

### Predictor Training

When training the methods, we followed the principles for systematic ML method training presented earlier (Niroula and Vihinen, 2016). We started by choosing the ML algorithm. Three ML algorithms were tested based on our earlier experience in variation interpretation. We implemented predictors with LGBM, RF, and XGBoost and performed 10-fold cross-validation (CV) Table 2. The methods were trained on all the features. Because the two versions of gradient boosting were somewhat better than when using RF, we chose LGBM as it is faster. The OPM was 0.67, accuracy 0.88, and MCC 0.75. Further, the predictors were quite balanced. Due to the use of GOSS and EFB technologies, LightGBM was the fastest to train and test.

**TABLE 2:** Comparison of method performance in 10-fold cross-validation when using all the features for training. The numbers are averages.

Measure	RF	XGBoost	LGBM
TP	1,528	1,633.4	1,651.3
TN	2,307.9	2,364.3	2,343.1
FP	222.7	166.3	187.5
FN	498.7	393.3	375.4
PPV	0.87	0.91	0.90
NPV	0.82	0.86	0.86
Sensitivity	0.75	0.81	0.81
Specificity	0.91	0.93	0.93
Accuracy	0.84	0.88	0.88
MCC	0.68	0.75	0.75
OPM	0.59	0.67	0.67
AUC	0.83	0.87	0.87

Next, we performed feature selection. We collected 1,085 features, including 617 amino acid features, 436 variation type features, 25 neighborhood features, 2 evolutionary conservation features, 3 protein features, 1 Gene Ontology feature, and 1 functional annotation feature. RFE was used to recursively reduce the number of features. To decide the optimal number of features, we tested the performance in 10-fold CV with different numbers of features: all, 100, 50, 20, or 10. We wanted to proceed with the smallest possible number of features as the event space is large. The ratio of human, plant, and animal variations in the 10-fold CV for this purpose was 100:10:1.

Further, the methods were implemented with or without rejection and with or without GO features. Classification with the reject option was found useful in PON-P2 (Niroula et al., 2015) to distinguish the category for UVs and obtain reliable predictions for benign and pathogenic cases. UV variants cannot be classified as pathogenic or benign. This class also implies the heterogeneity of phenotypes in different individuals bearing the same variant and is a normal feature for certain variants.

The results of the performance assessment are in Supplementary Tables S1, S2. The performances are clearly better when using the GO feature and when applying the rejection option. The results overall are very similar within the different tests for different numbers of features indicating that the number of features can be substantially reduced without a major impact on the performance. The implementation without rejection and GO feature had the best performance with a predictor trained on 50 features (OPM, 0.479), but differences were minimal for methods trained with different numbers of features, effectively in the third decimal place (Supplementary Table S2). Similarly, the differences in the other measures were very small or non-existent.

GO features have been useful in several predictors, such as SNPs&GO (Capriotti et al., 2013) and PON-P2 (Niroula et al., 2015). However, GO annotations are far from complete, and the coverage in non-human organisms can be very low, or the annotations may be completely missing. Therefore, to facilitate as many predictions as possible, we developed methods both with and without GO features.

When using GO features (but without the rejection option) (Supplementary Table S1), the overall performance is substantially better than without GO details (Supplementary Table S2). The results for 50 features were the best (OPM, 0.673), but those for 20 features were very close (OPM, 0.671). The results for the other measures were also very close irrespective of the number of features, thus indicating that the number of features could be significantly reduced.

Without GO but with rejection, the best OPM was achieved with all features (OPM 0.671). However, differences are marginal in the third decimal. The results with the GO feature (Supplementary Table S1) but without rejection are close to those for methods with rejection but without GO annotations (OPM 0.676 without rejection). The performance is further increased when rejection is applied (OPM shifted from 0.812 to 0.832, MCC from 0.865 to 0.880). The coverage of predictions increased substantially when GO features were not used, typically by 20%, thus allowing predictions for many more variants. There is thus a balance between the number of cases that can be predicted and the optimal performance.

**FIGURE 1:**
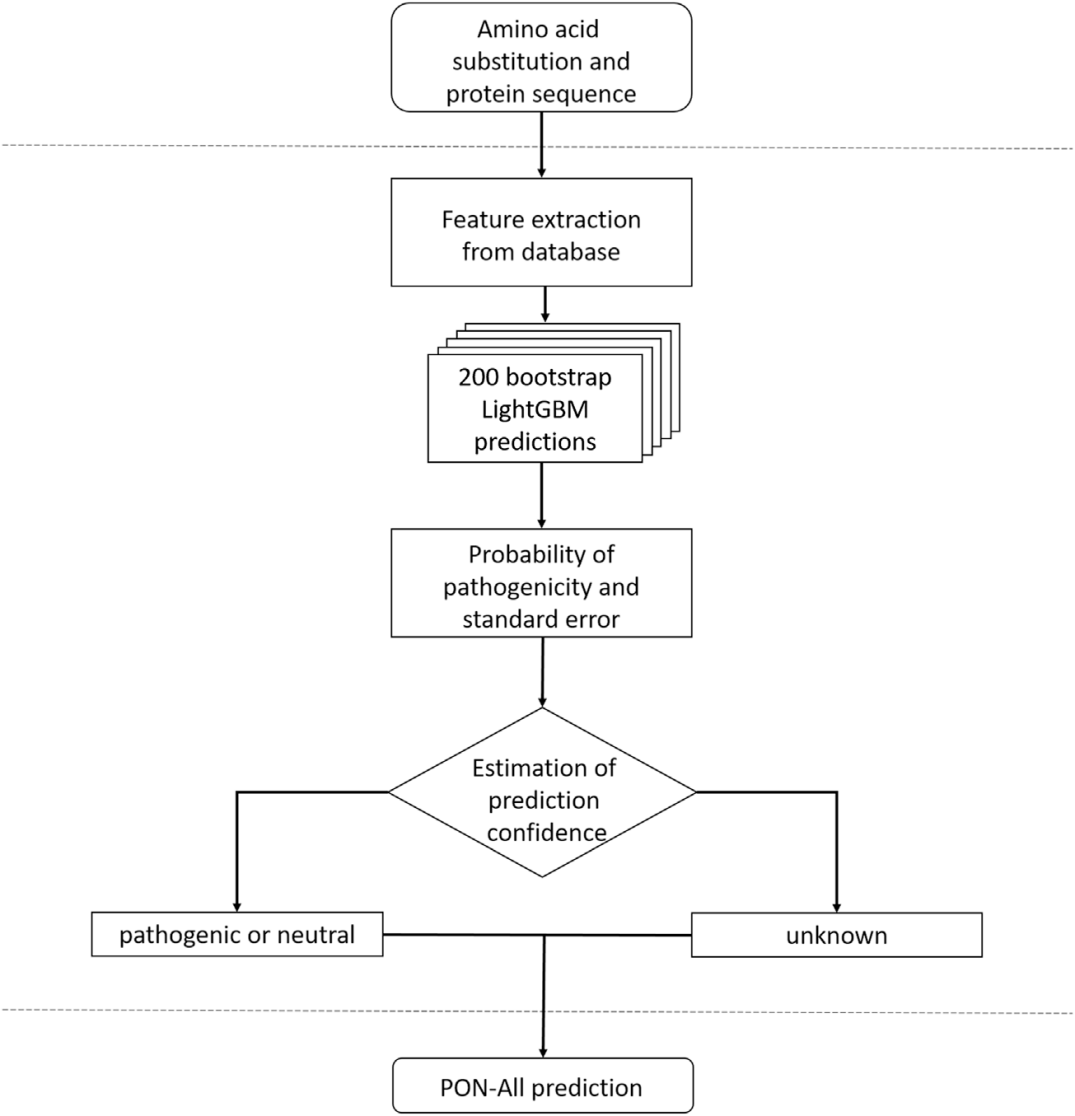
Flowchart for PON-All predictor.

Based on the results, we chose to train the final predictors with 20 features. It is beneficial to use a smaller set of features to better cover the event space (as it is smaller), thereby increasing representativeness and reducing the risk of overfitting. The flowchart of PON-All is shown in Figure 1. We trained two predictors, one with and one without GO terms. The selected features are listed in Supplementary Tables S3, S4. Of the 20 features on both lists, 15 were shared by the two methods. The selected features represent different types of features, including amino acid features, variation type and neighborhood features, evolutionary conservation features, and protein feature. The unique features in the method with GO annotations included amino acid propensities, neighborhood features, conservation feature, and GO annotations. In the method without GO annotations, the unique features were for amino acid features and neighborhood feature. The importance of the features is indicated in Supplementary Tables S3, S4. The protein feature is the most informative, followed by sums of log odd ratios for GO terms and functional site terms. Sequence conservation features, the number of homologs and SIFT 4G feature, are followed in significance by position within sequence and number of nonpolar amino acids. The other selected features have clearly lower significance in the case of the predictor with the GO feature. The highest scores for features in the case of prediction without GO feature are for protein feature, number of SwissProt homologs, position within a sequence, number of nonpolar amino acids, and SIFT 4G score.

We trained the final predictors with 20 features both when including and excluding GO annotations and named the tool PON-All because it can predict the effects of amino acid substitutions in proteins from any organism, unlike many existing methods. By default, predictions are made using GO features. However, if the annotations are missing, a predictor not requiring these features is used.

### Performance Assessment With Blind Test Data Set

The performance of the method was tested with the blind test set, data that were withdrawn in the initial partitioning and not used during method development. Table 3 shows results for PON-All with and without GO annotations. There are results for the entire test data set and separately for the three groups of organisms. As the ratios of variations in the groups are widely different, it is important to look at them separately. Otherwise, the largest group, for human variations, would dominate the overall output.

**TABLE 3:** Performance assessment in the blind test set with and without the GO feature. The results are shown with and without (in brackets) rejection.

Measure	All variants		Humans		Animals		Plants	
	w GO	wo GO	w GO	wo GO	w GO	wo GO	w GO	wo GO
TP	1,945 (2,278)	1,201 (1,928)	1,274 (1,552)	789 (1,327)	72 (102)	64 (112)	603 (624)	341 (489)
TN	1,855 (2,284)	1,344 (2,109)	1,421 (1,780)	1,052 (1,659)	118 (143)	98 (140)	318 (361)	201 (310)
FP	143 (365)	177 (540)	138 (326)	148 (447)	4 (26)	14 (29)	4 (13)	15 (64)
FN	217 (433)	251 (783)	94 (268)	154 (493)	35 (53)	12 (43)	88 (112)	85 (247)
PPV	0.932 (0.862)	0.872 (0.781)	0.902 (0.826)	0.842 (0.748)	0.947 (0.797)	0.821 (0.794)	0.993 (0.980)	0.958 (0.884)
NPV	0.895 (0.841)	0.843 (0.729)	0.938 (0.869)	0.872 (0.771)	0.771 (0.730)	0.891 (0.765)	0.783 (0.763)	0.703 (0.557)
Sensitivity	0.900 (0.840)	0.827 (0.711)	0.931 (0.853)	0.837 (0.729)	0.673 (0.658)	0.842 (0.723)	0.873 (0.848)	0.800 (0.664)
Specificity	0.928 (0.862)	0.884 (796)	0.911 (0.845)	0.877 (0.788)	0.967 (0.846)	0.875 (0.828)	0.988 (0.965)	0.931 (0.829)
Accuracy	0.913 (0.851)	0.856 (0.753)	0.921 (0.849)	0.859 (0.761)	0.830 (0.756)	0.862 (0.778)	0.909 (0.887)	0.844 (0.720)
MCC	0.827 (0.703)	0.712 (0.509)	0.841 (0.697)	0.714 (0.518)	0.678 (0.515)	0.714 (0.555)	0.817 (0.777)	0.695 (0.466)
AUC	0.913 (0.851)	0.856 (0.753)	0.921 (0.85)	0.855 (0.758)	0.818 (0.751)	0.858 (0.775)	0.929 (0.895)	0.842 (0.747)
OPM	0.763 (0.617)	0.628 (0.429)	0.781 (0.611)	0.630 (0.438)	0.588 (0.434)	0.631 (0.470)	0.751 (0.701)	0.608 (0.391)
Coverage	0.776 (1.000)	0.555 (1.000)	0.746 (1.000)	0.546 (1.000)	0.707 (1.000)	0.580 (1.000)	0.913 (1.000)	0.578 (1.000)

In the results for the entire data set and when using GO annotations, the OPM was 0.763, accuracy 0.913, and MCC 0.827. Overall, the method is well balanced (Table 3). Without GO features, the performance dropped somewhat, OPM to 0.628, accuracy to 0.856, and MCC to 0.712. The results for the option without rejection were further reduced. The overall coverage with GO and with rejection was 0.776 and without rejection complete (1.000). The corresponding figures for predictions without GO terms were 0.551 and 1.000. Thus, the increase in coverage comes with reduced overall performance.

ClinVar provides community-assessed variation information. It would have been interesting to train the tool with benign cases from this database, but there were only 370 cases. They were used for an additional test of specificity. After removing variants used for PON-All training, there were 298 variants left. The specificity for this data set was 0.982 with the GO feature and 0.84 without the GO parameter. The coverages were 0.729 and 0.515, respectively. The specificity is very similar to that of PON-P2 on a much larger ExAC data set (Niroula and Vihinen, 2019).

When we compared the results for variants in humans, animals, and plants separately (Table 3), predictions for humans were somewhat increased from those for all variants, OPM of 0.781 (vs. 0.763), accuracy of 0.921 (vs. 0.913), and MCC of 0.841 (vs. 0.827). The differences are about the same magnitude also for the other measures. The predictions are about the same degree lower for plants as they are increased for humans in comparison to the total. For example, the best results, those with GO features and rejection in plants, were for OPM 0.751 vs. 0.763 for all variants and, similarly, accuracy 0.909 vs. 0.913 and MCC 0.817 vs. 0.827. The corresponding measures for animals were substantially lower, 0.588, 0.830, and 0.678. The reason for the drop in the scores for animals is that only a small number of animal-specific variants were available. Overall, the results for PON-All were good, and the tool can be used for reliable predictions of unknown cases.

To further test the impact of data sets, we trained separate predictors for human, animal, and plant variants using the PON-All training data. The results of the blind test are shown in Supplementary Table S5. The performance scores for humans and plants are close to those for PON-All. Interestingly, the performance of the human-specific predictor is slightly lower than for PON-All. OPM in a blind test with GO is 0.774 while the figure for PON-All is 0.781, the corresponding figures for accuracy are 0.918 and 0.921 and for MCC 0.836 and 0.841. Similarly, all the other scores are also very close to those for PON-All. Thus, the differences are very small in the third decimal. Similar observations were made with plant variants.

The coverage is also slightly lower for the human-specific prediction, whereas the specific predictor has somewhat higher coverage in plants. The coverage of the animal-specific predictor is clearly lower (0.605 vs. 0.707) than the results for PON-All. The training data for animal variants was so small that this is expected. What is somewhat unexpected is that the scores are better for the specific than the generic predictor. MCC of the specific tool with GO feature and rejection is 0.803 *versus* 0.678. Similarly, accuracy is 0.903 *versus* 0.830 and OPM 0.734 *versus* 0.588. One could have expected human variants to increase the performance of animal cases, but that seems not to be the case.

Even the results for animal variant predictors are promising, especially when considering that only 306 variants were used for training. The blind test set for animals contained 324 variants. In conclusion, the performances of the PON-All were close to those for specific predictors, and since the generic predictor has been trained with a large number of cases, the method can predict the effects of variants in all kinds of proteins in all organisms. PON-All was slightly better for human and plant variants. Only in the case of the animal variants, the specific tool was somewhat better. In conclusion, the generic PON-All is overall the best choice. We would argue this to be true also in the case of animal variants, as the large body of cases for humans will allow details for predictions in animals, as well. However, this may be species-dependent.

The largest portion of variations were for humans. Most previous methods that can be used for other organisms have been trained on human data only. Therefore, we tested the performance when animal and plant variants were predicted with a human-specific predictor. The results are in Supplementary Table S6. Compared to generic PON-All (Table 3), in the case of animal variants, some scores are better for animal data. In the case of plant variants, the generic predictor provides better results. These results can be explained mainly by the small number of available animal variants. Human cases provide additional strength for the prediction. Plants are so different from humans that a similar effect is not seen. The coverage of animal variants with human-specific predictors is somewhat smaller than for PON-All and substantially reduced for plant variants. Comparison to animal and plant-specific predictors in Supplementary Table S5 indicates that the measures for the human-based predictions are clearly lower, except for coverage of animal variants. There, the wider distribution of human training cases leads to increased performance.

### Comparison to Other Tools

It was not possible to compare the performance to non-human variant predictors because they are not available as predictors or they are based on the same data sets as used herein. The method for mammalian variants (Plekhanova et al., 2019) is an ML tool chosen among several tested algorithms. The data set contained human, mouse, dog, and cattle variants. Fourteen features without selection were used. Two methods have been described for plant variants. One of them is specific for *A. thaliana* (Kono et al., 2018) and was trained on the same data as PON-All. The method is based on the likelihood ratio test implemented with the BAD_Mutations pipeline (Kono et al., 2016). The other plant predictor was trained on *Arabidopsis* cases (Kovalev et al., 2018) using transfer learning based on 18 features but without feature selection.

We compared the performance of PON-All to several widely used generic variant tolerance predictors. The compared tools included CADD (Kircher et al., 2014), FATHMM (Rogers et al., 2018), MetaLR and MetaSVM (Dong et al., 2015), MutationTaster (Schwarz et al., 2014), PolyPhen2 (1), PON-P2 (Niroula et al., 2015), PROVEAN (Choi et al., 2012), and SIFT 4G (Vaser et al., 2016). Table 4 indicates that, with the GO feature, the scores are better than for PON-P2, which has the closest performance. The other methods have clearly lower performance. These results are in line with many previous benchmarks that have shown PON-P2 to be the best or among the best tools (Niroula and Vihinen, 2019; Orioli and Vihinen, 2019; Sarkar et al., 2020). The coverage of PON-All is almost 20% higher than that for PON-P2, thus providing a significant improvement when also the performance scores are improved.

**TABLE 4:** Blind test performance of PON-All compared to other predictors.

	PON-all wGO	PON-all woGO	PON-P2	SIFT 4G	PolyPhen2	MutationTaster	FATHMM	PROVEAN	MetaSVM	MetaLR	CADD_10^a^	CADD_15^a^	CADD_20^a^
TP	1,274	789	831	1,391	1,530	1,544	1,113	1,364	1,239	1,234	1,630	1,599	1,545
TN	1,421	1,052	1,032	1,197	1,003	1,044	1,472	1,320	1,651	1,639	498	710	1,020
FP	138	148	141	591	790	771	323	490	166	178	1,319	1,107	797
FN	94	154	132	288	149	135	563	313	440	445	49	80	134
PPV	0.902	0.842	0.855	0.702	0.659	0.667	0.775	0.736	0.882	0.874	0.553	0.591	0.660
NPV	0.938	0.872	0.887	0.806	0.871	0.885	0.723	0.808	0.790	0.786	0.910	0.899	0.884
Sens	0.931	0.837	0.863	0.828	0.911	0.920	0.664	0.813	0.738	0.735	0.971	0.952	0.920
Spes	0.911	0.877	0.880	0.669	0.559	0.575	0.820	0.729	0.909	0.902	0.274	0.391	0.561
ACC	0.921	0.859	0.872	0.746	0.730	0.741	0.745	0.770	0.827	0.822	0.609	0.660	0.734
MCC	0.841	0.714	0.742	0.503	0.500	0.523	0.491	0.543	0.659	0.649	0.337	0.410	0.512
OPM	0.781	0.63	0.661	0.423	0.416	0.436	0.414	0.459	0.570	0.559	0.291	0.341	0.426
Coverage	0.746	0.546	0.544	0.883	0.884	0.890	0.884	0.888	0.890	0.890	0.890	0.890	0.890

a For CADD, 10,15, and 20 are three common thresholds.

In the case of CADD, results are provided for three widely used thresholds since the developers did not optimize the threshold. By putting the value to 20, it was possible to increase the performance. However, this came with the cost of increased false-positive hits. As previous benchmarks have indicated (Niroula and Vihinen, 2019; Orioli and Vihinen, 2019), CADD has a substantial false hit rate so that about 1/3 of benign variants are classified as pathogenic. PON-P2, MetaSVM, and MetaLR had the best performances after PON-All (Table 4). PON-P2 is the closest to PON-All. The scores for MetaSVM and MetaLR are clearly lower.

### Example of Application

To highlight the applicability and performance of the PON-All tool, we predicted all possible amino acid substitutions in three related proteins. Predictions were made for Bruton tyrosine kinase (BTK) pleckstrin homology (PH) domains. The sequences were obtained from UniProtKB for human (Q06187-1), mouse (P35991-1), and *Drosophila melanogaster* (P08630-1) BTK. The sequences were aligned with Clustal Omega (Sievers et al., 2011). Harmful variants in human BTK cause X-linked agammaglobulinemia (XLA), a primary immunodeficiency (Mohamed et al., 2009), in mouse X-linked immunodeficiency (Khan et al., 1995). In *Drosophila*, the related protein, BTK29A, is involved, for example, in survival and male genital development (Hamada et al., 2005). Numerous XLA-causing variants are known in humans and listed in BTKbase (Väliaho et al., 2006). In xid mice, variant E41K in the PH domain is the causative alteration. *Drosophila fic* ^*p*^ variant is due to intronic alteration and causes alternative splicing and deletion of the PH domain (Baba et al., 1999). Thus, variants in the BTK PH domain are related to important functions in all the three organisms; thereby, it is of interest to investigate the effects of variants in these domains.

**FIGURE 2:**
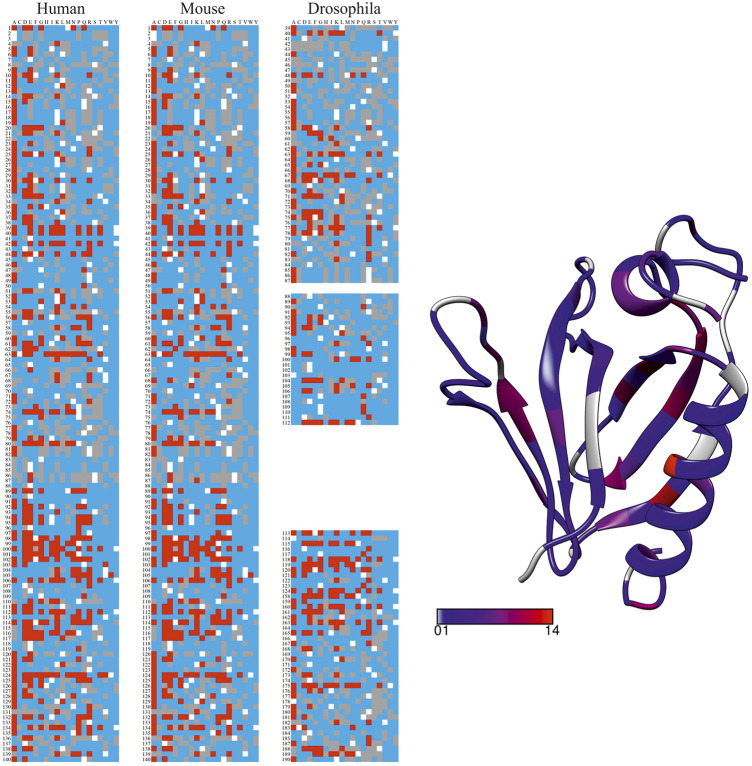
Visualization of PON-All predictions (without GO terms) for human, mouse, and *Drosophila* BTK PH domains. The sequences were adjusted based on multiple sequence alignment so that corresponding amino acid positions in the three proteins are on the same line. Red indicates predicted pathogenic variation, benign variants are blue, UVs are gray, and the original residue is white. The distribution of the number of pathogenic variants is shown to the right in the human PH domain (PDB 6tt2). The color scheme for the numbers of pathogenic variants is the scale in the figure.

All the 19 possible single amino acid substitutions in each position were generated and predicted with PON-All. The results are shown in Figure 2, where the predicted pathogenic and benign variants are color-coded. Mouse and *Drosophila* sequences were aligned with human BTK by either deleting amino acids or adding empty lines to keep the sequences in alignment. The human BTK PH domain (PDB id 6tt2) to the right indicates the number of predicted harmful variants by a rainbow coloring scheme. The maximum number of harmful variants in a position was 14, shown in bright red. These residues are in the middle of secondary structural elements. The majority of the variants in tolerant positions, gray for those where no harmful variants were predicted and blue with small numbers of harmful variants, are mainly in the ends of secondary structural elements and in surface loops. Interestingly, positions 7 and 8 in the middle of the first β-strand tolerate all substitutions. The differences in vulnerabilities are also clearly visible in the graphs for the mouse and fruit fly sequences.

The method facilitates the first-time systematic comparison of site vulnerabilities for sequences from various organisms.

### PON-All Web Application

PON-All is freely available as a web application at http://structure.bmc.lu.se/PON-All/ and http://8.133.174.28:8999/. The program has a user-friendly web interface that accepts variations in protein sequence, as amino acid substitutions, or in a VCF file (human). Batch submission, including all variants and proteins of interest, is accepted and recommended. PON-All provides a complete report, which is sent to the user by email when ready.

## Discussion

We have developed the first generic variant pathogenicity predictor that has been trained and tested on variants also from animals and plants. PON-All shows good performance for the prediction of the three types of organisms. Because the number of animal variants was clearly smaller than that for plants or humans, the drop in the performance is understandable. We could have increased animal variants by including cases from rodent databases. However, we felt that it would have biased the data as lots of these variants are generated to model human conditions. Overfitting is a potential problem in gradient boosting methods. Independent cross-validation and blind test set results are well in line. If the method were overfitted, there would be discrepancies in the performance for the different data sets and partitions. Further, we have used extensive data set and a minimal number of features, which are the classical remedies for overfitting.

PON-All has improved performance in comparison to the other methods. In addition to higher reliability, the tool has also increased coverage, up to 20% in comparison to PON-P2. This is important and facilitates reliable predictions in substantially increased numbers. These methods will never reach 100% coverage because disease-causing variants display a continuum. Some variants can be disease-related in some individuals, but not in all who carry the variant. PON-All is good at recognizing such cases and ranking them as UVs.

The use of GO features and the reject option clearly improved the performance. This is the default mode of prediction; however, apart from human and some well studied model organisms not a feasible option. GO annotations are scarce or missing for less investigated organisms. Even in these cases, predictions are still rather reliable. The coverage of such variants was reduced. Still, the new method makes a significant contribution also in these cases.

Fifteen out of the 20 features per predictor are shared with the tools that have been trained with or without GO features. Thus, in addition to the GO feature, some others differ between the two installations. This indicates the interplay between features and that it is important to perform proper feature selection. Some of the previous tools have been developed without feature selection, just using all the features that were originally collected. When the numbers of variants are small, as in this study, especially for animals, the event space remains very large if feature selection is not applied. A small number of training and test cases cannot cover such a space, and the representativeness is low (Schaafsma and Vihinen, 2018).

Methods like this are used for variant interpretation and recognition of pathogenic or, more generally, harmful variants. PON-All can be used for all organisms. In the case of variants in pathogens, it has to be remembered that harmful variants in such organisms mean harmful variants for that organism, not for human or other target organisms.

## Data Availability

The data sets presented in this study can be found in online repositories. The names of the repository/repositories and accession number(s) can be found below: VariBench http://structure.bmc.lu.se/VariBench and http://8.133.174.28:8999/.

## References

[B1] Adzhubei I. A. Schmidt S. Peshkin L. Ramensky V. E. Gerasimova A. Bork P. (2010). A Method and Server for Predicting Damaging Missense Mutations. Nat. Methods 7, 248–249. 10.1038/nmeth0410-248 20354512 PMC2855889

[B2] Ali H. Urolagin S. Gurarslan Ö. Vihinen M. (2014). Performance of Protein Disorder Prediction Programs on Amino Acid Substitutions. Hum. Mutat. 35, 794–804. 10.1002/humu.22564 24753228

[B3] Altschul S. Madden T. L. Schaffer A. A. Zhang J. Zhang Z. Miller W. (1997). Gapped BLAST and PSI-BLAST: a New Generation of Protein Database Search Programs. Nucleic Acids Res. 25, 3389–3402. 10.1093/nar/25.17.3389 9254694 PMC146917

[B4] Baba K. Takeshita A. Majima K. Ueda R. Kondo S. Juni N. (1999). The Drosophila Bruton's Tyrosine Kinase (Btk) Homolog Is Required for Adult Survival and Male Genital Formation. Mol. Cell Biol. 19, 4405–4413. 10.1128/mcb.19.6.4405 10330180 PMC104399

[B5] Breiman L. (2001). Random Forests. Mach. Learn. 45, 5–32. 10.1023/a:1010933404324

[B6] Buchfink B. Xie C. Huson D. H. (2015). Fast and Sensitive Protein Alignment Using DIAMOND. Nat. Methods 12, 59–60. 10.1038/nmeth.3176 25402007

[B7] Calabrese R. Capriotti E. Fariselli P. Martelli P. L. Casadio R. (2009). Functional Annotations Improve the Predictive Score of Human Disease-Related Mutations in Proteins. Hum. Mutat. 30, 1237–1244. 10.1002/humu.21047 19514061

[B8] Capriotti E. Calabrese R. Fariselli P. Martelli P. L. Altman R. B. Casadio R. (2013). WS-SNPs&GO: a Web Server for Predicting the Deleterious Effect of Human Protein Variants Using Functional Annotation. BMC Genomics 14 (Suppl. 3), S6. 10.1186/1471-2164-14-S3-S6 PMC3665478 23819482

[B9] Carbon S. Ireland A. Mungall C. J. Shu S. Marshall B. Lewis S. (2009). AmiGO: Online Access to Ontology and Annotation Data. Bioinformatics 25, 288–289. 10.1093/bioinformatics/btn615 19033274 PMC2639003

[B10] Chen T. Guestrin C. XGBoost (2016). A Scalable Tree Boosting System. KDD '16: Proceedings of the 22nd ACM SIGKDD International Conference on Knowledge Discovery and Data Mining. San Fransisco, CA, USA: ACM, 785–794.

[B11] Choi Y. Sims G. E. Murphy S. Miller J. R. Chan A. P. (2012). Predicting the Functional Effect of Amino Acid Substitutions and Indels. PLoS One 7, e46688. 10.1371/journal.pone.0046688 23056405 PMC3466303

[B12] Dong C. Wei P. Jian X. Gibbs R. Boerwinkle E. Wang K. (2015). Comparison and Integration of Deleteriousness Prediction Methods for Nonsynonymous SNVs in Whole Exome Sequencing Studies. Hum. Mol. Genet. 24, 2125–2137. 10.1093/hmg/ddu733 25552646 PMC4375422

[B13] Guyon I. Weston J. Barnhill S. Vapnik V. (2002). Gene Selection for Cancer Classification Using Support Vector Machines. Mach. Learn. 46, 389–422. 10.1023/a:1012487302797

[B14] Hamada N. Bäckesjö C.-M. Smith C. I. E. Yamamoto D. (2005). Functional Replacement ofDrosophilaBtk29A with Human Btk in Male Genital Development and Survival. FEBS Lett. 579, 4131–4137. 10.1016/j.febslet.2005.06.042 16023106

[B15] Kaminker J. S. Zhang Y. Waugh A. Haverty P. M. Peters B. Sebisanovic D. (2007). Distinguishing Cancer-Associated Missense Mutations from Common Polymorphisms. Cancer Res. 67, 465–473. 10.1158/0008-5472.can-06-1736 17234753

[B16] Kawashima S. Kanehisa M. (2000). AAindex: Amino Acid Index Database. Nucleic Acids Res. 28, 374. 10.1093/nar/28.1.374 10592278 PMC102411

[B17] Ke G. Meng Q. Finley T. Wang T. Chen W. Ma W. (2017). A Highly Efficient Gradient Boosting Decision Tree Neural Information Processing Systems. USA: La Jolla, CA.

[B18] Khan W. N. Alt F. W. Gerstein R. M. Malynn B. A. Larsson I. Rathbun G. (1995). Defective B Cell Development and Function in Btk-Deficient Mice. Immunity 3, 283–299. 10.1016/1074-7613(95)90114-0 7552994

[B19] Kircher M. Witten D. M. Jain P. O'Roak B. J. Cooper G. M. Shendure J. (2014). A General Framework for Estimating the Relative Pathogenicity of Human Genetic Variants. Nat. Genet. 46, 310–315. 10.1038/ng.2892 24487276 PMC3992975

[B20] Kono T. J. Y. Fu F. Mohammadi M. Hoffman P. J. Liu C. Stupar R. M. (2016). The Role of Deleterious Substitutions in Crop Genomes. Mol. Biol. Evol. 33, 2307–2317. 10.1093/molbev/msw102 27301592 PMC4989107

[B21] Kono T. J. Y. Lei L. Shih C.-H. Hoffman P. J. Morrell P. L. Fay J. C. (2018). Comparative Genomics Approaches Accurately Predict Deleterious Variants in Plants. G3 (Bethesda) 8, 3321–3329. 10.1534/g3.118.200563 30139765 PMC6169392

[B22] Kovalev M. S. Igolkina A. A. Samsonova M. G. Nuzhdin S. V. (2018). A Pipeline for Classifying Deleterious Coding Mutations in Agricultural Plants. Front. Plant Sci. 9, 1734. 10.3389/fpls.2018.01734 30546376 PMC6279870

[B23] Landrum M. J. Lee J. M. Riley G. R. Jang W. Rubinstein W. S. Church D. M. (2014). ClinVar: Public Archive of Relationships Among Sequence Variation and Human Phenotype. Nucl. Acids Res. 42, D980–D985. 10.1093/nar/gkt1113 24234437 PMC3965032

[B24] Lockwood S. Krishnamoorthy B. Ye P. (2011). Neighborhood Properties Are Important Determinants of Temperature Sensitive Mutations. PLoS One 6, e28507. 10.1371/journal.pone.0028507 22164302 PMC3229608

[B25] Mohamed A. J. Yu L. Bäckesjö C.-M. Vargas L. Faryal R. Aints A. (2009). Bruton's Tyrosine Kinase (Btk): Function, Regulation, and Transformation with Special Emphasis on the PH Domain. Immunol. Rev. 228, 58–73. 10.1111/j.1600-065x.2008.00741.x 19290921

[B26] Munoz-Torres M. Carbon S. (2017). Get GO! Retrieving GO Data Using AmiGO, QuickGO, API, Files, and Tools. Methods Mol. Biol. 1446, 149–160. 10.1007/978-1-4939-3743-1_11 27812941

[B27] Nair P. S. Vihinen M. , (2013). VariBench: A Benchmark Database for Variations. Hum. Mutat. 34, 42–49. 10.1002/humu.22204 22903802

[B28] Nicholas F. W. (2003). Online Mendelian Inheritance in Animals (OMIA): a Comparative Knowledgebase of Genetic Disorders and Other Familial Traits in Non-laboratory Animals. Nucleic Acids Res. 31, 275–277. 10.1093/nar/gkg074 12520001 PMC165521

[B29] Niroula A. Urolagin S. Vihinen M (2015). PON-P2: Prediction Method for Fast and Reliable Identification of Harmful Variants. PLoS ONE 10 (2), e0117380. 10.1371/journal.pone.0117380 25647319 PMC4315405

[B30] Niroula A. Vihinen M. (2019). How Good Are Pathogenicity Predictors in Detecting Benign Variants? PLoS Comput. Biol. 15, e1006481. 10.1371/journal.pcbi.1006481 30742610 PMC6386394

[B31] Niroula A. Vihinen M (2016). PON-mt-tRNA: a Multifactorial Probability-Based Method for Classification of Mitochondrial tRNA Variations. Nucleic Acids Res. 44, 2020–2027. 10.1093/nar/gkw046 26843426 PMC4797295

[B32] Niroula A. Vihinen M. (2017). Predicting Severity of Disease-Causing Variants. Hum. Mutat. 38, 357–364. 10.1002/humu.23173 28070986

[B33] Niroula A. Vihinen M. (2016). Variation Interpretation Predictors: Principles, Types, Performance, and Choice. Hum. Mutat. 37, 579–597. 10.1002/humu.22987 26987456

[B34] Olatubosun A. Väliaho J. Härkönen J. Thusberg J. Vihinen M. Pon-P. (2012). Integrated Predictor for Pathogenicity of Missense Variants. Hum. Mutat. 33, 1166–1174. 10.1002/humu.22102 22505138

[B35] Orioli T. Vihinen M. (2019). Benchmarking Subcellular Localization and Variant Tolerance Predictors on Membrane Proteins. BMC Genomics 20, 547. 10.1186/s12864-019-5865-0 31307390 PMC6631444

[B36] Pavey T. G. Gilson N. D. Gomersall S. R. Clark B. Trost S. G. (2017). Field Evaluation of a Random Forest Activity Classifier for Wrist-Worn Accelerometer Data. J. Sci. Med. Sport 20, 75–80. 10.1016/j.jsams.2016.06.003 27372275

[B37] Pedregosa F. Varoquaux G. Gramfort A. Michel V. Thirion B. Grisel O. (2011). Scikit-learn: Machine Learning in python. J. Mach. Learn. Res. 12, 2825–2830.

[B38] Plekhanova E. Nuzhdin S. V. Utkin L. V. Samsonova M. G. (2019). Prediction of Deleterious Mutations in Coding Regions of Mammals with Transfer Learning. Evol. Appl. 12, 18–28. 10.1111/eva.12607 30622632 PMC6304693

[B39] Richards S. Aziz N. Bale S. Bick D. Das S. Gastier-Foster J. (2015). Standards and Guidelines for the Interpretation of Sequence Variants: a Joint Consensus Recommendation of the American College of Medical Genetics and Genomics and the Association for Molecular Pathology. Genet. Med. 17, 405–424. 10.1038/gim.2015.30 25741868 PMC4544753

[B40] Rogers M. F. Shihab H. A. Mort M. Cooper D. N. Gaunt T. R. Campbell C. (2018). FATHMM-XF: Accurate Prediction of Pathogenic Point Mutations via Extended Features. Bioinformatics 34, 511–513. 10.1093/bioinformatics/btx536 28968714 PMC5860356

[B41] Sarkar A. Yang Y. Vihinen M. (2020). Variation Benchmark Datasets: Update, Criteria, Quality and Applications. Database 2020, baz117. 32016318 10.1093/database/baz117 PMC6997940

[B42] Schaafsma G. C. P. Vihinen M. (2018). Representativeness of Variation Benchmark Datasets. BMC Bioinforma. 19 (1), 461. 10.1186/s12859-018-2478-6 PMC6267811 30497376

[B43] Schwarz J. M. Cooper D. N. Schuelke M. Seelow D. (2014). MutationTaster2: Mutation Prediction for the Deep-Sequencing Age. Nat. Methods 11, 361–362. 10.1038/nmeth.2890 24681721

[B44] Shen B. Vihinen M. (2004). Conservation and Covariance in PH Domain Sequences: Physicochemical Profile and Information Theoretical Analysis of XLA-Causing Mutations in the Btk PH Domain. Protein Eng. Des. Sel. 17, 267–276. 10.1093/protein/gzh030 15082835

[B45] Shomer B. (1997). Seqalert-a Daily Sequence Alertness Server for the EMBL and SWISSPROT Databases. Bioinformatics 13, 545–547. 10.1093/bioinformatics/13.5.545 9367127

[B46] Sievers F. Wilm A. Dineen D. Gibson T. J. Karplus K. Li W. (2011). Fast, Scalable Generation of High‐quality Protein Multiple Sequence Alignments Using Clustal Omega. Mol. Syst. Biol. 7, 539. 10.1038/msb.2011.75 21988835 PMC3261699

[B47] Väliaho J. Smith C. I. E. Vihinen M. (2006). BTKbase: the Mutation Database for X-Linked Agammaglobulinemia. Hum. Mutat. 27, 1209–1217. 16969761 10.1002/humu.20410

[B48] Vaser R. Adusumalli S. Leng S. N. Sikic M. Ng P. C. (2016). SIFT Missense Predictions for Genomes. Nat. Protoc. 11, 1–9. 10.1038/nprot.2015.123 26633127

[B49] Vihinen M. (2012). How to Evaluate Performance of Prediction Methods? Measures and Their Interpretation in Variation Effect Analysis. BMC Genomics 13 (Suppl. 4), S2. 10.1186/1471-2164-13-S4-S2 PMC3303716 22759650

[B50] Vihinen M. (2020). Problems in Variation Interpretation Guidelines and in Their Implementation in Computational Tools. Mol. Genet. Genomic Med. 8, e1206. 10.1002/mgg3.1206 32160417 PMC7507483

[B51] Vihinen M. (2013). Guidelines for Reporting and Using Prediction Tools for Genetic Variation Analysis. Hum. Mutat. 34, 275–282. 10.1002/humu.22253 23169447

[B52] Vihinen M. (2017). How to Define Pathogenicity, Health, and Disease? Hum. Mutat. 38, 129–136. 10.1002/humu.23144 27862583

[B53] Wang D. Zhang Y. Zhao Y. LightGBM (2017). Proceedings of the 2017 International Conference on Computational Biology and Bioinformatics - ICCBB. October 18 - 20, 2017. New York, NY, United States. Association for Computing Machinery, 7–11.

[B54] Yang Y. Urolagin S. Niroula A. Ding X. Shen B. Vihinen M. (2018). PON-tstab: Protein Variant Stability Predictor. Importance of Training Data Quality. Int. J. Mol. Sci. 19, 19. 10.3390/ijms19041009 PMC5979465 29597263

[B55] Yang Y. Zeng L. Vihinen M. (2021). Prediction of Effects of Variants on Protein Solubility. Int. J. Mol. Sci. 22, 8027. 34360790 10.3390/ijms22158027 PMC8348231

[B56] Yang Y. Niroula A. Shen B. Vihinen M. (2016). PON-sol: Prediction of Effects of Amino Acid Substitutions on Protein Solubility. Bioinformatics 32, 2032–2034. 10.1093/bioinformatics/btw066 27153720

[B57] Yu B. Qiu W. Chen C. Ma A. Jiang J. Zhou H. (2020). SubMito-XGBoost: Predicting Protein Submitochondrial Localization by Fusing Multiple Feature Information and eXtreme Gradient Boosting. Bioinformatics 36, 1074–1081. 10.1093/bioinformatics/btz734 31603468

[B58] Zhang J. Mucs D. Norinder U. Svensson F. LightGBM (2019). LightGBM: An Effective and Scalable Algorithm for Prediction of Chemical Toxicity-Application to the Tox21 and Mutagenicity Data Sets. J. Chem. Inf. Model. 59, 4150–4158. 10.1021/acs.jcim.9b00633 31560206

