# Supplementary material for: PON-All: Amino Acid Substitution Tolerance Predictor for All Organisms
Source: Front Mol Biosci. 2022 Jun 16;9:867572. doi: 10.3389/fmolb.2022.867572 (PMC9245922; doi:10.3389/fmolb.2022.867572)
Supplement: Supplementary file 1 [file DataSheet1.docx]

Supplementary Material

# Supplementary Tables

Supplementary Table 1. LightGBM 10-fold CV performance with GO feature. The first number is for a method with rejection option and the numbers in brackets for a method without rejection.

| Measure | all features | 100 features | 50 features | 20 features | 10 features |
| --- | --- | --- | --- | --- | --- |
| TP | 1295.9(1651.3) | 1299.1(1651) | 1301.5(1652.3) | 1296(1647.8) | 1265.1(1620.4) |
| TN | 2031.6(2343.1) | 2041.8(2343.3) | 2047.1(2346.4) | 2049.6(2345) | 2049.2(2342) |
| FP | 59.7(187.5) | 60.5(187.3) | 60.3(184.2) | 57.7(185.6) | 61.8(188.6) |
| FN | 143.6(375.4) | 144.1(375.7) | 147.3(374.4) | 155.9(378.9) | 168(406.3) |
| PPV | 0.956(0.898) | 0.955(0.898) | 0.955(0.900) | 0.957(0.899) | 0.953(0.896) |
| NPV | 0.934(0.862) | 0.934(0.862) | 0.933(0.863) | 0.930(0.862) | 0.925(0.853) |
| Sensitivity | 0.899(0.814) | 0.899(0.814) | 0.897(0.815) | 0.891(0.813) | 0.881(0.799) |
| Specificity | 0.971(0.926) | 0.971(0.926) | 0.971(0.927) | 0.973(0.927) | 0.971(0.926) |
| Accuracy | 0.942(0.876) | 0.942(0.876) | 0.942(0.877) | 0.940(0.876) | 0.935(0.869) |
| MCC | 0.880(0.750) | 0.880(0.750) | 0.878(0.752) | 0.875(0.750) | 0.865(0.736) |
| AUC | 0.938(0.872) | 0.938(0.871) | 0.938(0.873) | 0.936(0.872) | 0.930(0.864) |
| OPM | 0.832(0.671) | 0.832(0.671) | 0.830(0.673) | 0.826(0.671) | 0.812(0.655) |
| Coverage | 0.775(1.000) | 0.778(1.000) | 0.780(1.000) | 0.781(1.000) | 0.778(1.000) |

Supplementary Table 2. LightGBM 10-fold CV performance without GO feature. The first number is for a predictor with rejection option and the numbers in brackets for a method without rejection.

| Measure | all features | 100 features | 50 features | 20 features | 10 features |
| --- | --- | --- | --- | --- | --- |
| TP | 789.3(1400.5) | 804.1(1395) | 807.5(1395) | 808.6(1384.6) | 767.2(1340.6) |
| TN | 1443.5(2177.1) | 1470(2178.4) | 1482.5(2182.1) | 1505.3(2183.5) | 1498.9(2166) |
| FP | 83.5(353.5) | 85.4(352.2) | 86.4(348.5) | 89.9(347.1) | 95.3(364.6) |
| FN | 188.2(626.2) | 194.2(631.7) | 199.1(631.7) | 222.6(642.1) | 252.1(686.1) |
| PPV | 0.904(0.798) | 0.903(0.798) | 0.903(0.800) | 0.899(0.799) | 0.889(0.786) |
| NPV | 0.885(0.777) | 0.884(0.775) | 0.882(0.776) | 0.871(0.773) | 0.856(0.760) |
| Sensitivity | 0.807(0.691) | 0.805(0.688) | 0.801(0.688) | 0.783(0.683) | 0.752(0.661) |
| Specificity | 0.945(0.860) | 0.945(0.861) | 0.945(0.862) | 0.944(0.863) | 0.940(0.856) |
| Accuracy | 0.891(0.785) | 0.890(0.784) | 0.889(0.785) | 0.881(0.783) | 0.867(0.769) |
| MCC | 0.770(0.563) | 0.768(0.561) | 0.765(0.563) | 0.749(0.559) | 0.718(0.531) |
| AUC | 0.869(0.776) | 0.869(0.774) | 0.868(0.775) | 0.860(0.773) | 0.846(0.759) |
| OPM | 0.696(0.479) | 0.694(0.477) | 0.691(0.479) | 0.671(0.475) | 0.638(0.450) |
| Coverage | 0.550(1.000) | 0.560(1.000) | 0.565(1.000) | 0.576(1.000) | 0.573(1.000) |

Supplementary Table 3. Features used to train PON-All with GO feature.

| Importance | Name | Description | Score | Reference |
| --- | --- | --- | --- | --- |
| 1 | sequence len | protein sequence length | 613 |  |
| 2 | LR | Sum of log odd ratios for GO terms | 573 |  |
| 3 | sift4G hits | number of homologs SwissProt | 350 | (1) |
| 4 | Functional Site | sum of log odd ratios for functional site terms | 268 |  |
| 5 | mut_residue | position within sequence | 187 |  |
| 6 | sift4G scores | SIFT4G scores in SwissProt | 166 | (1) |
| 7 | DOSZ010103 | amino acid similarity matrix based on the THREADER force field | 100 | (2) |
| 8 | FUKS010101 | Surface composition of amino acids in intracellular proteins of thermophiles (percent) | 87 | (3) |
| 9 | NonPolarAA | number of nonpolar amino acids | 73 |  |
| 10 | AA20D.P | number of P in 20-dimensional vector for neighborhood | 68 | (4) |
| 11 | FASG760101 | Molecular weight | 67 | (5) |
| 12 | AA20D.G | number of G in 20-dimensional vector for neighborhood | 64 | (4) |
| 13 | AA20D.A | number of A in 20-dimensional vector for neighborhood | 55 | (4) |
| 14 | SUYM030101 | linker propensity index | 54 | (6) |
| 15 | KOSJ950106 | context-dependent optimal substitution matrices for buried beta | 52 | (7) |
| 16 | AA20D.S | number of S in 20-dimensional vector for neighborhood | 49 | (4) |
| 17 | AA20D.I | number of I in 20-dimensional vector for neighborhood | 49 | (4) |
| 18 | KOSJ950114 | context-dependent optimal substitution matrices for buried residues | 48 | (7) |
| 19 | AA20D.F | number of F in 20-dimensional vector for neighborhood | 44 | (4) |
| 20 | DOSZ010101 | amino acid similarity matrix based on the sausage force field | 33 | (2) |

Supplementary Table 4. Features used to train PON-All without GO feature.

| Importance | Name | Description | Score | Reference |
| --- | --- | --- | --- | --- |
| 1 | sequence len | protein sequence length | 840 |  |
| 2 | sift4G hits | number of homologs in SwissProt | 571 | (1) |
| 3 | FASG760101 | molecular weight | 291 | (5) |
| 4 | mut_residue | position within sequence | 137 |  |
| 5 | DOSZ010101 | amino acid similarity matrix based on the sausage force field | 125 | (2) |
| 6 | SUYM030101 | linker propensity index | 111 | (6) |
| 7 | NonPolarAA | number of nonpolar amino acids | 85 |  |
| 8 | AA20D.P | number or P in 20-dimensional vector for neighborhood | 83 | (4) |
| 9 | OVEJ920104 | environment-specific amino acid substitution matrix for accessible residues | 76 | (8) |
| 10 | sift4G scores | SIFT4G scores in SwissProt | 74 | (1) |
| 11 | AA20D.G | number of G in 20-dimensional vector for neighborhood | 73 | (4) |
| 12 | AA20D.A | number of A in 20-dimensional vector for neighborhood | 72 | (4) |
| 13 | FITW660101 | mutation values for the interconversion of amino acid pairs | 68 | (9) |
| 14 | OVEJ920102 | environment-specific amino acid substitution matrix for alpha residues | 62 | (8) |
| 15 | AA20D.C | number of C in 20-dimensional vector for neighborhood | 62 | (4) |
| 16 | KUMS000103 | distribution of amino acid residues in the alpha-helices in thermophilic proteins | 61 | (10) |
| 17 | AA20D.S | number of S in 20-dimensional vector for neighborhood | 57 | (4) |
| 18 | AA20D.F | number of F in 20-dimensional vector for neighborhood | 53 | (4) |
| 19 | AA20D.I | number of I in 20-dimensional vector for neighborhood | 52 | (4) |
| 20 | AURR980119 | normalized positional residue frequency at helix termini C | 47 | (11) |

Supplementary Table 5. Results for methods trained specifically for human, animal and plant variants. Details are provided for training and blind test performance assessment, with and without GO features. The results are shown with and without (in brackets) rejection.

| Measure | Human | | | | Animals | | | | Plants | | | |
| --- | --- | --- | --- | --- | --- | --- | --- | --- | --- | --- | --- | --- |
|  | Training-wGO | Training-woGO | BT-wGO^a^ | BT-woGO | Training-wGO | Training-woGO | BT-wGO | BT-woGO | Training-wGO | Training-woGO | BT-wGO | BT-woGO |
| TP | 1023.4(1375.8) | 619.8(1147.2) | 1248(1534) | 766(1285) | 10.3(13.4) | 6.5(13.4) | 109(134) | 64(125) | 245.4(251.4) | 175.1(224.2) | 606(625) | 469(625) |
| TN | 1900.5(2187.9) | 1454.4(2067.1) | 1401(1768) | 1052(1686) | 5.9(11) | 6.4(10.6) | 68(130) | 76(133) | 140.2(151.4) | 58(123.1) | 322(362) | 143(273) |
| FP | 49.9(172.1) | 64.3(292.9) | 131(338) | 130(420) | 0.9(3.4) | 1(3.8) | 15(39) | 14(36) | 1.1(4.8) | 8.5(33.1) | 3(12) | 40(101) |
| FN | 144.6(374.6) | 201.4(603.2) | 105(286) | 166(535) | 0.5(2.8) | 0.3(2.8) | 4(21) | 8(30) | 3.6(8.7) | 7.9(55.9) | 99(111) | 20(111) |
| PPV | 0.953(0.889) | 0.905(0.796) | 0.905(0.819) | 0.855(0.754) | 0.911(0.794) | 0.868(0.750) | 0.879(0.775) | 0.821(0.776) | 0.995(0.981) | 0.954(0.870) | 0.995(0.981) | 0.921(0.861) |
| NPV | 0.930(0.855) | 0.879(0.774) | 0.930(0.861) | 0.864(0.759) | 0.936(0.774) | 0.968(0.794) | 0.944(0.861) | 0.905(0.816) | 0.975(0.945) | 0.878(0.771) | 0.765(0.765) | 0.877(0.711) |
| Sensitivity | 0.874(0.786) | 0.754(0.655) | 0.922(0.843) | 0.822(0.706) | 0.963(0.815) | 0.929(0.796) | 0.965(0.865) | 0.889(0.806) | 0.985(0.966) | 0.957(0.862) | 0.860(0.849) | 0.959(0.849) |
| Specificity | 0.974(0.927) | 0.958(0.876) | 0.914(0.840) | 0.890(0.801) | 0.880(0.740) | 0.850(0.742) | 0.819(0.769) | 0.844(0.787) | 0.993(0.969) | 0.874(0.788) | 0.991(0.968) | 0.781(0.730) |
| Accuracy | 0.937(0.867) | 0.886(0.782) | 0.918(0.841) | 0.860(0.757) | 0.925(0.797) | 0.907(0.785) | 0.903(0.815) | 0.864(0.796) | 0.988(0.968) | 0.934(0.834) | 0.901(0.889) | 0.911(0.809) |
| MCC | 0.866(0.728) | 0.747(0.775) | 0.836(0.681) | 0.715(0.510) | 0.844(0.561) | 0.805(0.540) | 0.803(0.635) | 0.729(0.593) | 0.974(0.931) | 0.831(0.646) | 0.804(0.781) | 0.769(0.575) |
| AUC | 0.921(0.860) | 0.856(0.767) | 0.918(0.842) | 0.856(0.753) | 0.924(0.794) | 0.917(0.769) | 0.903(0.815) | 0.864(0.795) | 0.989(0.969) | 0.916(0.825) | 0.918(0.909) | 0.874(0.790) |
| OPM | 0.814(0.647) | 0.672(0.551) | 0.774(0.594) | 0.632(0.431) | 0.802(0.504) | 0.745(0.476) | 0.734(0.545) | 0.646(0.505) | 0.962(0.901) | 0.777(0.562) | 0.734(0.706) | 0.703(0.495) |
| Coverage | 0.759(1.000) | 0.569(1.000) | 0.734(1.000) | 0.538(1.000) | 0.546(1.000) | 0.464(1.000) | 0.605(1.000) | 0.500(1.000) | 0.938(1.000) | 0.599(1.000) | 0.931(1.000) | 0.605(1.000) |

^a^BT, blind test data set.

Supplementary Table 6. The performance of the method when trained on human data and tested on animal and plants variants. The results are shown with and without (in brackets) rejection.

| Measure | Plant | | Animal | |
| --- | --- | --- | --- | --- |
|  | w GO | wo GO | w GO | wo GO |
| TP | 286(472) | 158(389) | 69(95) | 54(108) |
| TN | 322(361) | 202(314) | 113(147) | 95(141) |
| FP | 1(13) | 15(60) | 2(22) | 12(28) |
| FN | 154(264) | 143(347) | 32(60) | 17(47) |
| PPV | 0.997(0.973) | 0.913(0.866) | 0.972(0.812) | 0.818(0.794) |
| NPV | 0.676(0.578) | 0.586(0.475) | 0.779(0.710) | 0.848(0.75) |
| Sensitivity | 0.65(0.641) | 0.525(0.529) | 0.683(0.613) | 0.761(0.697) |
| Specificity | 0.997(0.965) | 0.931(0.84) | 0.983(0.87) | 0.888(0.834) |
| ACC | 0.797(0.75) | 0.695(0.633) | 0.843(0.747) | 0.837(0.769) |
| MCC | 0.66(0.578) | 0.477(0.355) | 0.707(0.502) | 0.657(0.538) |
| OPM | 0.56(0.479) | 0.391(0.301) | 0.618(0.423) | 0.572(0.454) |
| Coverage | 0.687(1.000) | 0.467(1.000) | 0.667(1.000) | 0.549(1.000) |

**References**

[1] Vaser, R, Adusumalli, S, Leng, SN, Sikic, M, and Ng, PC, SIFT missense predictions for genomes. *Nat Protoc*. (2016) 11:1-9.

[2] Dosztányi, Z, and Torda, AE, Amino acid similarity matrices based on force fields. *Bioinformatics*. (2001) 17:686-99.

[3] Fukuchi, S, and Nishikawa, K, Protein surface amino acid compositions distinctively differ between thermophilic and mesophilic bacteria. *J Mol Biol*. (2001) 309:835-43.

[4] Lockwood, S, Krishnamoorthy, B, and Ye, P, Neighborhood properties are important determinants of temperature sensitive mutations. *PLoS One*. (2011) 6:e28507.

[5] Fasman, GD, Handbook of Biochemistry and Molecular Biology. in: G.D. Fasman, (Ed.), CRC Press, Cleveland, 1976.

[6] Suyama, M, and Ohara, O, DomCut: prediction of inter-domain linker regions in amino acid sequences. *Bioinformatics*. (2003) 19:673-4.

[7] Koshi, JM, and Goldstein, RA, Context-dependent optimal substitution matrices. *Protein Eng*. (1995) 8:641-5.

[8] Overington, J, Donnelly, D, Johnson, MS, Sali, A, and Blundell, TL, Environment-specific amino acid substitution tables: tertiary templates and prediction of protein folds. *Protein Sci*. (1992) 1:216-26.

[9] Fitch, WM, An improved method of testing for evolutionary homology. *J Mol Biol*. (1966) 16:9-16.

[10] Kumar, S, Tsai, CJ, and Nussinov, R, Factors enhancing protein thermostability. *Protein Eng*. (2000) 13:179-91.

[11] Aurora, R, and Rose, GD, Helix capping. *Protein Sci*. (1998) 7:21-38.
